# Individual and family level factors associated with physical and mental health-related quality of life among people living with HIV in rural China

**DOI:** 10.1186/s12889-018-6352-2

**Published:** 2019-01-03

**Authors:** Yongkang Xiao, Chunqing Lin, Li Li, Guoping Ji

**Affiliations:** 1 Department of HIV/AIDS Prevention, Anhui Provincial Center for Disease control and Prevention, Hefei, China; 2 Semel Institute for Neuroscience and Human Behavior, Center for Community Health, University of California at Los Angeles, Los Angeles, CA USA; 3 Anhui Provincial Center for Woman and Child Health, 38 Yonghong Road, Hefei, 230032 China

**Keywords:** HIV, Physical health, Mental health, Health-related quality of life, China

## Abstract

**Background:**

One of the major challenges faced by people living with HIV (PLH) is the compromised quality of life due to the negative impact of HIV on their health. HIV/AIDS control effort should go beyond laboratory and lay more emphasis on improving the health-related quality of life (HRQoL) for PLH. The objective of this study is to evaluate the physical and mental HRQoL of PLH in rural China, and explore the relationship between HRQoL and individual- and family-level factors.

**Methods:**

A cross-sectional study was conducted among 522 PLH in Anhui, China. Participant’s sociodemographic characteristics, family status, and HIV-related factors were collected. Physical health summary score (PHS) and mental health summary score (MHS) of quality of life were measured. Multiple linear regressions were conducted to estimate the association of the individual- and family-level factors with MHS and PHS.

**Results:**

Male were more likely to report a higher level of PHS and MHS than female (*β* = 0.123, *P* = 0.009; *β* = 0.150, *P* = 0.002). Age was significantly negatively associated with the PHS (*β* = − 0.232, *P*<0.001) when other variables were controlled. Family size remained negatively correlated with PHS (*β* = − 0.105, *P* = 0.021). Family annual income was significantly positively associated with PHS and MHS (*β* = 0.126, *P* = 0.003; *β* = 0.135, *P* = 0.002).

**Conclusions:**

Future intervention should be carefully tailored to the specific needs of sub-populations (such as female and older PLH) considering their physical and mental HRQoL conditions. More attention and care should be provided to PLH with left-behind children in the family.

## Background

The availability of antiretroviral therapy (ART) and advancement of primary care system have prolonged the lifespan of people living with HIV (PLH), and HIV has become a chronic illness [1]. However, one of the significant challenges faced by PLH is the compromised quality of life due to the negative impact of HIV on their physical health and mental health [2, 3]. Literature has suggested that suboptimal quality of life may in turn have adverse influences on the patients’ ART retention, medication adherence, and treatment outcomes [4, 5]. Therefore, HIV/AIDS control effort should go beyond objective measures such as CD4 count and viral load, and lay more emphasis on improving the health-related quality of life (HRQoL) of PLH.

Previous studies have identified multilevel factors associated with HRQoL of PLH. Social-demographic characteristics, such as gender, age, and education, were all found to be correlated with HRQoL [6]. Older age was independently associated with lower physical HRQoL for both genders [7]. Disease-related factors associated with HRQoL included stage and time since diagnosis, current CD4 count/viral load, and severity of HIV/AIDS-related symptoms [8, 9], and being on ART was associated with better physical and mental health [10]. Psychological factors, such as depression [11] and social support [6, 9], were also found to be correlated with lower HRQoL of PLH. In addition, family factors play an essential role in the HRQoL of PLH in Asia because of the cultural norms with strong family values [12]. Family income [13] and family support [14] were the associating factors identified in previous studies, as family presents a source of support for the individual with HIV/AIDS, contributing to their physical and mental well-being [5]. Therefore, the study of PLH’s HRQoL should focus more on family-related factors.

In China, there were 501,000 reported cases of PLH by the end of 2014, most of whom lived in rural areas [15]. However, few studies reported the HRQoL of PLH in rural China. Existing studies in China mainly focus on the relationship between HRQoL and the PLH’s individual level factors, such as age [16, 17], employment status [18], income [19], social support [19–23], CD4 count [16], depression [20] and stigma [23–25]. Recently, mental HRQoLwas found to be negatively associated with unprotected anal intercourse among Chinese men who have sex with men (MSM) [26]. Studies examining the association between HRQoL and family-related factors were scant in China. A few exceptions included Hidru and colleagues’ (2016) study among MSM with HIV/AIDS, which reported that family income has an influence on PLH’s HRQoL [27]. A study conducted in Nanning, China reported that the lower HRQoL among older Chinese PLH was due to their limited social networks and heavy dependence on family to cope with HIV infection and ageism [28]. Wu and colleagues (2015) studied HIV clinic attendees and reported that family stigma was inversely correlated with most of the HRQoL domains [23]. On the contrary, family support was found to contribute to PLH’s HRQoL [29, 30]. The study aimed to evaluate the physical and mental HRQoL of PLH in rural China, and explore the relationship between HRQoL and individual- and family-level factors. The findings will provide insights to develop strategies to improve the HRQoL for PLH in rural China.

## Methods

### Study sample

The study used baseline data from a randomized controlled trial for HIV-affected families in Anhui Province, China. Though the number of new HIV cases through sexual infection has rapidly increased in recent years, the proportion of PLH who were caused by contaminated commercial plasma donations in the last century was still dominant among the existing total PLH population in certain areas, especially in rural areas [31–34]. A total of 522 PLH were recruited in this study based on the following criteria: (1) being aged 18 or over; (2) being local residents; and (3) having a sero-negative family member and a child in the family.

### Data collection

Data were collected between October 2011 and March 2013 from 32 villages in four counties (Lixin, Funan, Yinzhou, and Linquan) of Anhui. The four counties were similar in terms of demographics and HIV epidemiological profiles. Detailed study procedure was documented elsewhere [35, 36]. This study used MOS-HIV questionnaires as the main instrument to measure the quality of life, the questionnaire has been widely used in HIV-related studies and previously published in Wu’s article [37, 38].

Written informed consent was obtained from the participants. The study was reviewed and approved by the institutional review boards of the Anhui Provincial Center for Disease Control and Prevention and the University of California, Los Angeles.

### Measures

#### Quality of life

Quality of life was assessed with the Medical Outcomes Study HIV Health Survey (MOS-HIV) [39], which has 11 subscales. With these subscales, the physical health summary score (PHS) and mental health summary score (MHS) were generated. According to the unified algorithm, the physical functioning, pain and role functioning subscales contributed most to PHS, the mental health, health distress, quality of life, and cognitive functioning subscales contributed most to MHS, and the vitality, general health, and social functioning subscales contributed partially to both summary scores. Scores for these subscales and two aggregate measures (PHS and MHS) were calculated following the instruction and ranged from 0 (worst) to 100 (best) [37]. The MOS-HIV has high internal consistency and test-retest reliability [40] and has been tested among Chinese population [41]. The internal consistency reliability of the 11 subscales in our sample ranged from 0.75 to 0.91.

#### Sociodemographic characteristics

The PLH participants’ sociodemographic characteristics, including age, gender, marital status, and education, were collected.

#### Family factors

Three family factors were measured based on the following questions: (1) Family size: “How many people are there in your family, including those who live with you?”, (2) Family annual income: “What is your total yearly family income (yuan)?”, and (3) Spouse’s HIV sero-status; combination of three questions: “What is the health status of your spouse? (Ill/Dead/Healthy)” “What is his/her illness? (HIV/AIDS or Other- Specify)” “What was the cause of death?”

#### HIV- related factors

Two HIV-related factors were assessed based on the following questions: (1) Time of HIV diagnosis: “When did you first know your HIV infection diagnosed by a doctor? (Specify month/year)”; (2) ART status, “Are you currently on ARV treatment?” (Yes or No).

### Statistical analysis

All analyses were performed using SAS 9.4 software (SAS Institue Inc., Cary, NC, USA). First, we calculated descriptive statistics and frequencies (i.e., frequency, mean, and standard deviation) to describe the participants’ demographic characteristics, family and HIV-related conditions, and MHS and PHS scores. Secondly, we compared MHS and PHS on every variable of interest among the participants using T-test or ANOVA to determine whether MHS and PHS were different among different subgroups. Finally, multiple linear regression models were fitted to estimate the association of factors with the MHS and PHS adjusting for control variables. MHS and PHS were treated as continuous outcome variables in the multiple linear regression models.

## Results

### Sample characteristics

A total of 522 participants were included in this study. The average age was 48.57 years (range: 22–73 years). Approximately 45% were male. The majority of participants (81.4%) were married or cohabiting, and 40.2% had no education. Approximately one third (36.7%) of the participants had less than 10,000 yuan (approximately USD 1450) annual family income, 87.5% had four or more members in the family, and 26.8% reported their spouses were also HIV positive. The majority of participants (86.0%) had diagnosed as HIV-positive for six years or above, and 92.7% were on ART (Table 1).

**Table 1:** PHS and MHS by background demographic variables

Variables	n (%)	Physical health summary score		Mental health summary score	
		Mean (SE)	*P*	Mean (SE)	*P*
Gender					
Female	288 (55.2)	34.54(9.31)	<0.001	34.67(8.65)	0.001
Male	234(44.8)	37.24(8.99)		37.98(8.79)	
Age groups (year)					
< 45	192(36.8)	38.45(9.46)	<0.001	37.37(9.08)	3.079
45–54	182(34.9)	35.39(8.77)		35.70(9.06)	
≥ 55	148(28.4)	32.69(8.59)		35.12(8.19)	
Marital status					
Married/Cohabiting	425 (81.4)	36.14 (9.24)	0.042	36.44(8.84)	0.119
Divorced/Widowed/Other	97 (18.6)	34.03 (9.18)		34.89(8.91)	
Education					
Yes	312 (59.8)	36.94 (9.33)	< 0.001	37.43(8.83)	<0.001
None	210 (40.2)	33.99 (8.89)		34.26(8.59)	
Family size (people)					
3	65(12.5)	37.59(10.62)	0.094	36.26(9.74)	0.455
4–5	207(39.7)	36.12(9.47)		36.71(8.89)	
≥ 6	250(47.8)	34.96(8.64)		35.66(8.60)	
Family annual income (yuan)					
< 10,000	191(36.7)	34.78(9.12)	0.020	35.06(8.39)	0.008
10,000–19,999	177(34.0)	35.41(8.92)		35.84(9.33)	
≥ 20,000	152(29.2)	37.51(9.59)		37.97(8.68)	
Spouse’s HIV status					
HIV−/death/other	382(73.2)	36.23(9.42)	0.049	36.69(9.20)	0.013
HIV+	140(26.8)	34.43(8.72)		34.69(7.70)	
Diagnosis time (year)					
<3	23(4.4)	36.43(12.97)	0.252	34.96(9.80)	0.553
3–5	50(9.6)	37.97(10.38)		37.48(9.64)	
6–8	277(53.3)	35.19(8.95)		35.83(8.92)	
≥ 9	170(32.7)	35.95(8.83)		36.46(8.47)	
ART condition					
On ART	484(92.7)	35.33(9.06)	<0.001	35.88(8.83)	0.012
Not on ART	38(7.3)	41.14(10.13)		39.62(8.69)	

### HRQoL summary scores (PHS and MHS)

The mean score of PHS was 35.75 ± 9.26 (range: 12.10–62.97), and the mean score of MHS was 36.15 ± 8.86 (range: 8.24–60.92). Univariate analysis indicated that the mean scores of PHS and MHS were both different among groups by gender, education, family annual income, spouse’s HIV status, and ART status (all *P* < 0.05). In addition, PHS scores were significantly lower among older participants. PLH who were married/cohabiting reported significantly higher PHS score than those who were single, divorced, or widowed (*P* < 0.05) (Table 1).

### Factors associated with HRQoL summary scores (PHS and MHS)

All nine candidate variables were included in the multiple linear regression models. Five variables (gender, age, family size, family annual income, and ART condition) were significantly associated with PHS score in the final model. Male were more likely to report a higher level of PHS than female (*β* = 0.123; *P* = 0.009). Age was significantly negatively associated with the PHS (*β* = − 0.232; *P*<0.001) when other variables were controlled. Family size remained negatively correlated with PHS (*β* = − 0.105; *P* = 0.021). Family annual income was significantly positively associated with PHS (*β* = 0.126; *P* = 0.003). PLH who were not on ART reported better PHS than those who were on ART while other variables were held constant (*β* = 0.141; *P* = 0.001) (Table 2).

**Table 2:** The multiple linear regression analysis results of PHS and MHS

Model	Physical health summary score (*n* = 518)		Mental health summary score (n = 518)	
	Standardized *β*	*P*	Standardized *β*	*P*
Gender (ref. Female)	0.123	0.009	0.150	0.002
Age	−0.232	<0.001	−0.087	0.066
Marital status (ref. Married/Cohabiting)	−0.026	0.562	−0.044	0.329
Education (ref. Yes)	−0.023	0.635	−0.063	0.201
Family size	−0.105	0.021	−0.052	0.265
Family annual income	0.126	0.003	0.135	0.002
Spouse HIV status (ref. HIV−/death/other)	−0.082	0.065	−0.087	0.056
Diagnosis time	0.048	0.253	0.061	0.157
ART condition (ref. on ART)	0.141	0.001	0.098	0.021

Three variables, gender, family annual income, and ART status, were significantly associated with MHS in the final model. Male were also more likely to report a higher level of MHS than female (*β* = 0.150; *P* = 0.002). Family annual income was significantly positively associated with MHS (*β* = 0.135; *P* = 0.002). PLH who were not on ART reported better MHS than those who were on ART in the model (*β* = 0.098; *P* = 0.021) (Table 2).

## Discussion

In this study, two sociodemographic factors were found to be associated with HRQoL, including gender and age. Previous research on the relation between gender and HRQoL of PLH had mixed results. Some studies reported that mental HRQoL of male PLH was better than that of female PLH [42], while others generated opposite results [8, 13]. Other research showed no apparent correlation between gender and HRQoL [10]. This study showed that male PLH in rural China had better physical and mental HRQoL than their female counterparts, and it may be due to cultural differences. China has historically been a male-dominated country, and female PLH may bear more burden originated from social position and role orientation [43, 44]. Age is also a major factor that affects PLH’s physiological functioning. Natural aging of body organs can lead to declined body functions. Various HIV/AIDS comorbidities, such as tuberculosis, tumor, hepatitis, and/or opportunistic infections, all severely compromise the HRQoL of PLH [45, 46]. In this study, older age was associated with lower physical HRQoL. This finding is consistent with reports of other studies [13, 42, 47, 48]. However, age is associated with declining physical health, but not necessarily mental health. The association between age and MHS was not statistically significant in regression analysis, which suggests that older PLH may not always have worse mental health compared to younger PLH. Hence, researchers should carefully consider tailored approaches to address the differentiated health needs of specific sub-population (such as female and older PLH) to improve their HRQoL.

China has a distinct family-centered culture and custom, that most PLH receive or require support from their families [49, 50]. In addition, China’s law stipulated that grown-up children are duty-bound to support their parents. It is a common practice for generations of Chinese to live in the same household, especially in rural areas. Therefore, the family forms a crucial safety umbrella for PLH, and family members are the primary caregivers for daily care, treatment, psychosocial and financial support, and childcare [25]. We found that several family characteristics, including family size and family annual income, were associated with PLH’s HRQoL. PLH’s physical health was lower among those who had larger families, which could be explained by two possible reasons: first, the more members in a family, the fewer shares of limited family resources for every member; second, due to urbinazation in the last three decades, a large number of young adults migrate to major cities to seek higher income, leaving behind their young child (ren) to the elder parents. We speculated that the families of PLH in the study mainly consisted of grandchildren of PLH, who could not provide family support and at the same time increase child-care burden of PLH. The finding indicates that more service and care should be provided to PLH with left-behind children in the family. The associations between higher income and better HRQoL was supported by many previous studies, as good family economic condition means better living conditions and better health care for PLH [13, 42, 47, 48]. Thus, one of the important mechanisms to improve HRQoL of PLH in rural areas is to carry out effective strategies for poverty alleviation, which need a collaborative effort of the whole society.

Another family status deserves attention is spousal HIV status, that having an HIV-positive spouse was marginally associated with lower physical and mental HRQoL. In rural China, the spouse is usually the primary source of caring and support for PLH [42, 51]. Spouse’s HIV infection can to a great extent undermine the daily life care, material support, and psychological comfort a family can provide to PLH [52]. In addition, when a family has more than one HIV positive member, the burden of the family will often be more than doubled due to the escalated societal stigma and shame [53, 54]. Hence, HIV seroconcordant couples are in greater need of care and support to cope with aggravating impact of HIV.

Interestingly, current ART use was found to be associated with lower levels of physical and mental health. The finding should be interpreted with caution. At the time of the study, the test-and-treat policy has not been implemented in China, and ART was only provided to PLH with a CD4 lower than 500 (cells/mm3) and/or those with clinical symptoms [35]. The lower level of HRQoL found in PLH who were under ART was due to the advanced disease stages [47, 55, 56].

### Limitations

Some limitations of the study should be interpreted. First, we cannot draw causal inferences based on the cross-sectional data. Second, because the participants were recruited from Anhui province in China, findings may not be generalizable to PLH in other areas. Finally, the self-report data may be subject to socio-desirability bias.

## Conclusions

The study added to the current understanding of levels of HRQoL among PLH and the individual- and family-level factors associated with physical and mental HRQoL. The findings identified the family-related factors had a strong relationship on HRQoL of PLH in rural China. The results may have implications for intervention design and future research. Future intervention should be carefully tailored to the specific needs of sub-populations considering their physical and mental HRQoL conditions. For example, PLH with left-behind children in the family would need greater care and support. More intervention efforts should be paid to HIV seroconcordant couples to help them cope with aggravating impact of HIV.

## Abbreviations

ART: Antiretroviral therapy
CAPI: computer-assisted personal interview
HIV: Human immunodeficiency virus
HRQoL: Health-related quality of life
MHS: Mental health summary
MOS-HIV: Medical outcomes study HIV health survey
PHS: Physical health summary
PLH: People living with HIV
